# Feasibility of the chick chorioallantoic membrane model for preclinical studies on tumor radiofrequency ablation

**DOI:** 10.1186/s41747-023-00368-3

**Published:** 2023-09-25

**Authors:** Joel Wessendorf, Michael Scheschenja, Moritz B. Bastian, Alexander M. König, Axel Pagenstecher, Frederik Helmprobst, Malte Buchholz, Marina Tatura, Jarmila Jedelská, Andreas H. Mahnken

**Affiliations:** 1 Department of Diagnostic and Interventional Radiology, University Hospital Marburg, Philipps-University Marburg, Marburg, Germany; 2 Mouse Pathology and Electron Microscopy — Core Facility, Institute of Neuropathology, Philipps-University Marburg, Marburg, Germany; 3 Clinic for Gastroenterology, Endocrinology, Metabolism and Infectiology, Philipps-University Marburg, Marburg, Germany; 4 Small Animal MRI — Core Facility, Center for Tumor Biology and Immunology (ZTI), Philipps-University Marburg, Marburg, Germany

**Keywords:** Chickens, Chorioallantoic membrane, Neuroendocrine tumors, Radiofrequency ablation, Radiology (interventional)

## Abstract

**Background:**

We evaluated the feasibility of a chick chorioallantoic membrane (CAM) tumor model for preclinical research on tumor radiofrequency ablation (RFA).

**Methods:**

Fertilized chicken eggs were incubated and divided into five cohorts: RFA for 30 s (*n* = 5), RFA for 60 s (*n* = 5), RFA for 120 s (*n* = 4), sham (*n* = 8), and controls (*n* = 6). Xenografting using pancreatic neuroendocrine tumor cells of the BON-1 cell line was performed on embryonic day (ED) 8. The RFA was performed on ED 12. Survival, stereomicroscopic observations, and histological observations using hematoxylin–eosin (H&E) and Ki67 staining were evaluated.

**Results:**

The survival rates in the 30-s, 60-s, and 120-s, sham and control cohort were 60%, 60%, 0%, 100%, and 50%, respectively. Signs of bleeding and heat damage were common findings in the evaluation of stereomicroscopic observations. Histological examination could be performed in all but one embryo. Heat damage, bleeding, thrombosis, and leukocyte infiltration and hyperemia were regular findings in H&E-stained cuts. A complete absence of Ki67 staining was recorded in 33.3% and 50% of embryos in the 30-s and 60-s cohorts that survived until ED 14, respectively.

**Conclusions:**

The CAM model is a feasible and suiting research model for tumor RFA with many advantages over other animal models. It offers the opportunity to conduct *in vivo* research under standardized conditions. Further studies are needed to optimize this model for tumor ablations in order to explore promising but unrefined strategies like the combination of RFA and immunotherapy.

**Relevance statement:**

The chick chorioallantoic membrane model allows *in vivo* research on tumor radiofrequency ablation under standardized conditions that may enable enhanced understanding on combined therapies while ensuring animal welfare in concordance with the “Three Rs.”

**Key points:**

• The chorioallantoic membrane model is feasible and suiting for tumor radiofrequency ablation.

• Radiofrequency ablation regularly achieved reduction but not eradication of Ki67 staining.

• Histological evaluation showed findings comparable to changes in humans after RFA.

• The chorioallantoic membrane model can enable studies on combined therapies after optimization.

**Graphical Abstract:**

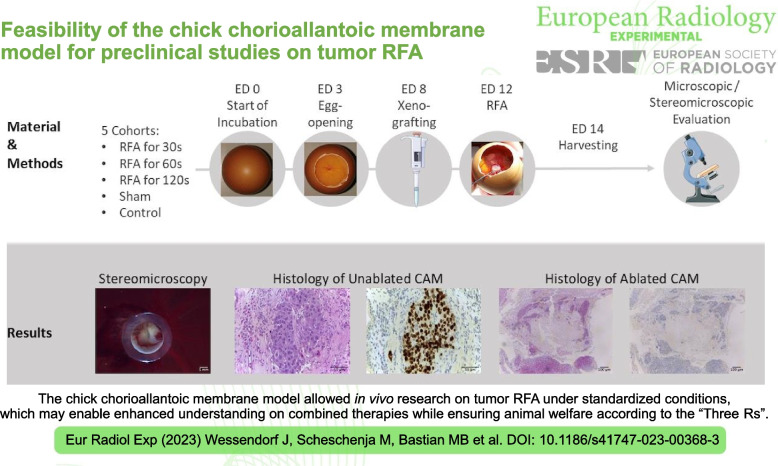

## Background

Thermoablative therapy methods like radiofrequency ablation (RFA) are known to be safe and effective alternatives to surgical therapy in different tumor entities, such as hepatocellular carcinoma [1, 2] and renal cell carcinoma [3] where thermoablative therapies are commonly used. Furthermore, RFA is also proven to be clinically effective in combination with transarterial (chemo)embolization in the therapy of human hepatocellular carcinoma and renal cell carcinoma [4, 5]. An important advantage of thermoablative methods over surgery is a lower risk for procedure-associated complications due to their minimal-invasive character and the possibility to perform these procedures under local anesthesia [6]. Therefore, minimal-invasive treatment options like RFA are already increasingly used in clinical practice as suiting alternatives for patients with increased surgical risk. Preclinical research on ablative strategies is especially important to explore promising but unrefined strategies like the combination of RFA and immunotherapy. But first, there is a need for a suitable and feasible preclinical research model for tumor RFA.

A preclinical research model for tumor ablations should enable repeated procedures under standardized conditions. Furthermore, the research model should be in line with the Three Rs of “The Principles of Humane Experimental Technique” — replacement, reduction, and refinement — to ensure animal welfare [7]. Commonly used animal models for RFA are murine and porcine models [8–10]. Problems with these models are that they are models of generally sentient animals, not always easily reproducible and/or cost-effective.

The chorioallantoic membrane (CAM) model is a non-sentient animal model that uses the CAM of the chick, which is created by the fusion of mesodermal layers of allantois and chorion [11, 12]. The CAM model is already established as a well-suited preclinical research model for a multitude of studies including oncologic and pharmacologic research [11, 13–16]. The innate and adaptive immunity of the chick are not fully developed until embryonic day (ED) 16 and ED 18, respectively [13, 17]. This lack of immunity in the first EDs allows xenografting of cells of different origins such as human cancer cells [18, 19]. Therefore, it provides a platform for the preclinical evaluation of different therapeutic strategies.

The aim of this study is to establish the CAM model as a feasible model for preclinical research on thermoablative tumor therapies like RFA.

## Methods

All experimentations performed on CAM model in this prospective study were in accordance with the EU directive 2010/63/EU for animal experiments and therefore did not require approval by an ethics committee [20].

### Cultivation and preparation for xenografting

**Fig. 1:**
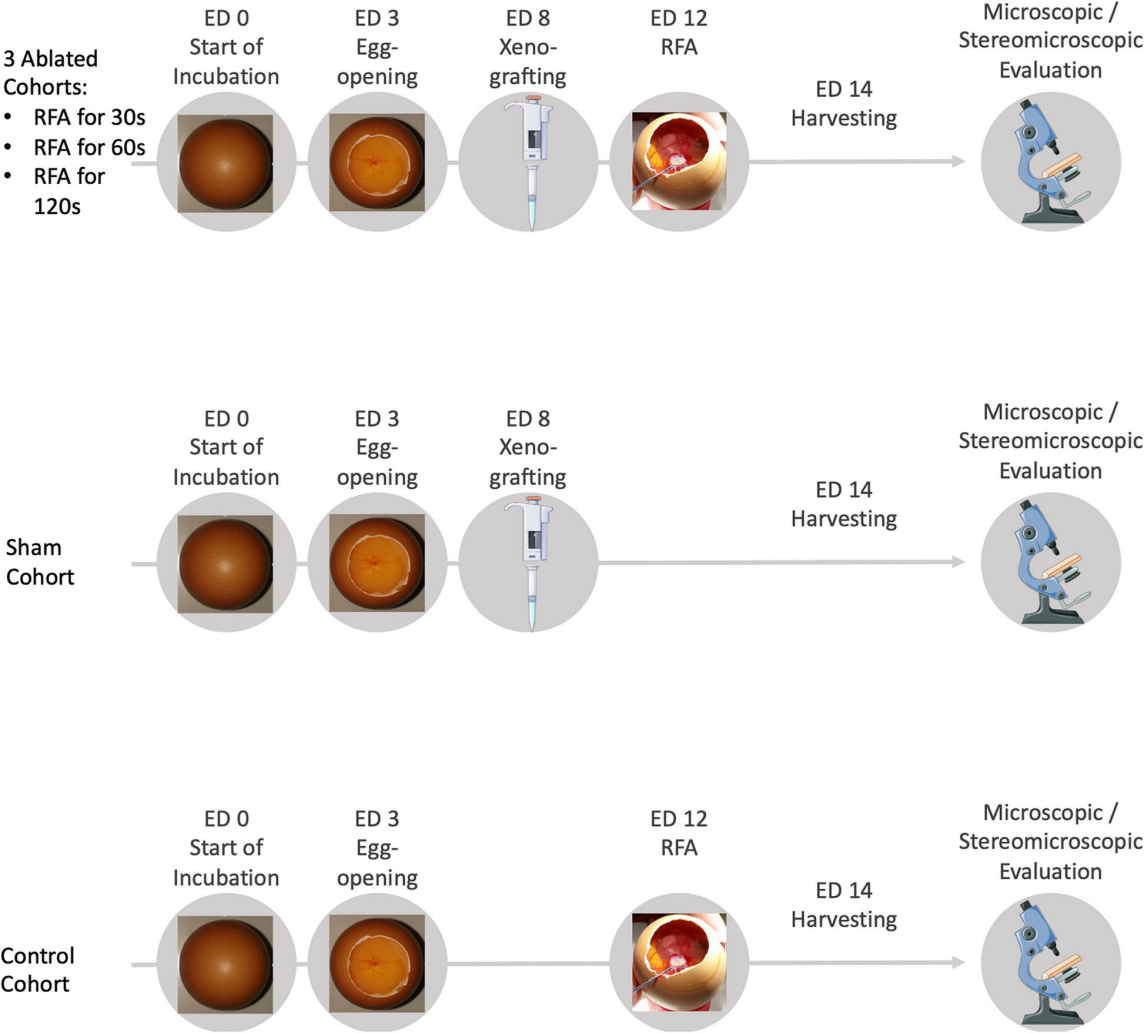
Overview of methods. Parts of the figure were drawn using pictures from Servier Medical Art (smart.servier.com), provided by Servier, licensed under a Creative Commons Attribution 3.0 Unported License (https://creativecommons.org/licenses/by/3.0/). *ED* Embryonic day, *RFA* Radiofrequency ablation

After delivery, fertilized chicken eggs (Brormann GmbH & Co. KG, Rheda-Wiedenbrück, Germany) were allowed to stay at room temperature for temperature equilibrium at least for 2 h. Subsequently, they were cleaned with 70% (v/v) ethanol and incubated at 37.8 °C and 60−70% relative humidity in a hatching incubator (Brutmaschine Easy 150, J. Hemel Brutgeräte GmbH, Verl, Germany). On ED 3, the eggs were taken out of the incubator and cleaned again with 70% ethanol. The egg shell was cracked at the broad pole using a manual egg opener. The cracked egg shell as well as the adjacent membrane was carefully removed with the help of forceps. A round window with a diameter of approximately 3 cm was thus created. Viable embryos were identified by clear blood vessels and a beating heart [21]. The window was covered with a sterile petri dish, and the eggs were returned into the incubator where they were further incubated on a frame which kept them in upright position until further use [14]. An overview over the methods is presented in Fig. 1.

### Xenografting

Xenografting was performed on ED 8, after the CAM was gently injured using an aseptic lens tissue as previously described [22]. A silicon ring was placed directly on the injured area. The cell line used for xenografting was the BON-1 (CVCL_3985) cell line which was derived from a lymph node metastasis of a serotonin-producing neuroendocrine tumor of pancreas [23]. A suspension of BON-1 cells (0.6–0.8 × 10^6^ in 20-µL medium) was mixed with 20 μL of Matrigel (Corning Life Sciences, Amsterdam, the Netherlands). The mix of Matrigel and cell suspension was implanted into the silicone ring. The round window was covered again with a petri dish. The egg was placed back into the incubator. The vitality of the embryos was examined daily, and tumor growth was observed via stereomicroscopy.

### Radiofrequency ablation

On ED 12 (4 days after tumor engraftment), radiofrequency ablation was performed with a CELON Power System (Olympus, Hamburg, Germany). ED 12 was chosen as the day of ablation as it enables both sufficient time for tumor growth and follow-up. A bipolar needle electrode (CELON ProSurge micro, Olympus, Hamburg, Germany) was inserted outside the silicone ring, and the electrode tip was positioned under the center of the silicone ring. The eggs were divided into five cohorts: ablation for 30 s with 4 W (*n* = 5), ablation for 60 s with 4 W (*n* = 5), ablation for 120 s with 4 W (*n* = 4), a sham cohort without therapy (*n* = 8), and a control cohort without xenografting prior to RFA (total: *n* = 6; 30 s with 4 W: *n* = 2; 60 s with 4 W: *n* = 2; 120 s with 4 W: *n* = 2).

### Evaluation of the outcomes

Survival of the embryos after RFA as well as stereomicroscopic changes were observed directly after RFA, 2 h after RFA, 15 h after RFA, and 40 h after RFA. On ED 14 (2 days after RFA), the CAM tumors were harvested, fixated in phosphate-buffered 4% paraformaldehyde, and embedded in paraffin. Serial 3-μm paraffin sections were processed and routinely stained with hematoxylin–eosin (H&E). Immunohistochemistry (IHC) staining was performed with monoclonal mouse antihuman Ki67 antibodies (Dako, Ki67, Clone MIB-1) to mark proliferating cells [16, 24]. Since the antibody does not recognize chicken Ki67, complete ablation was assumed when there was an absence of Ki67 staining in the CAM of embryos that survived until ED 14. Stereomicroscopic changes and immunohistochemistry were evaluated by observation with a Leica EZ4 HD stereomicroscope (Leica, Wetzlar, Germany) and a Leica DM1000 LED microscope (Leica, Wetzlar, Germany), respectively. Effectiveness of ablation, survival, and stereomicroscopic changes were exploratively analyzed.

## Results

### Effectiveness of ablation

The completeness in the 30-s cohort and 60-s cohort was 33.3% and 50%, respectively. There were more Ki67-positive cells on CAMs of the sham cohort than on ablated CAMs. The outcomes of the ablations are displayed in Table 1.

**Table 1:** Summary of outcomes

Cohort	Completeness (based on Ki67 staining)	Survival (time of death)	Stereomicroscopic observation directly after radiofrequency ablation
30 s, 4 W (*n* = 5)	33.3%	60% (2 h, 15 h)	Signs of bleeding (100%) Signs of heat damage (100%)
60 s, 4 W (*n* = 5)	50%	60% (2 h, 15 h)	Signs of bleeding (80%) Signs of heat damage (100%)
120 s, 4 W (*n* = 4)	Not applicable (no survival)	0% (3 × 2 h, 2 × 15 h)	Signs of bleeding (100%) Signs of heat damage (100%)
Sham (*n* = 8)	Not applicable (no ablation, all Ki67 +)	100%	Not applicable
Control (*n* = 6)	Not applicable (no xenografting, all Ki67 −)	50% (3 × 15 h)	Signs of bleeding (66.7%) Signs of heat damage (83.3%) Vaporization (16.7%)

The completeness is not evaluable in the 120-s sham and control cohort because no embryo survived in the 120-s cohort, no radiofrequency ablation was performed in the sham cohort, and no xenografting was done in the control cohort

### Survival of the chick embryos

The survival rates of the chick embryos in the 30-s cohort, 60-s cohort, 120-s cohort, sham cohort, and control cohort were 60%, 60%, 0%, 100%, and 50%, respectively.

### Stereomicroscopic observations

**Fig. 2:**
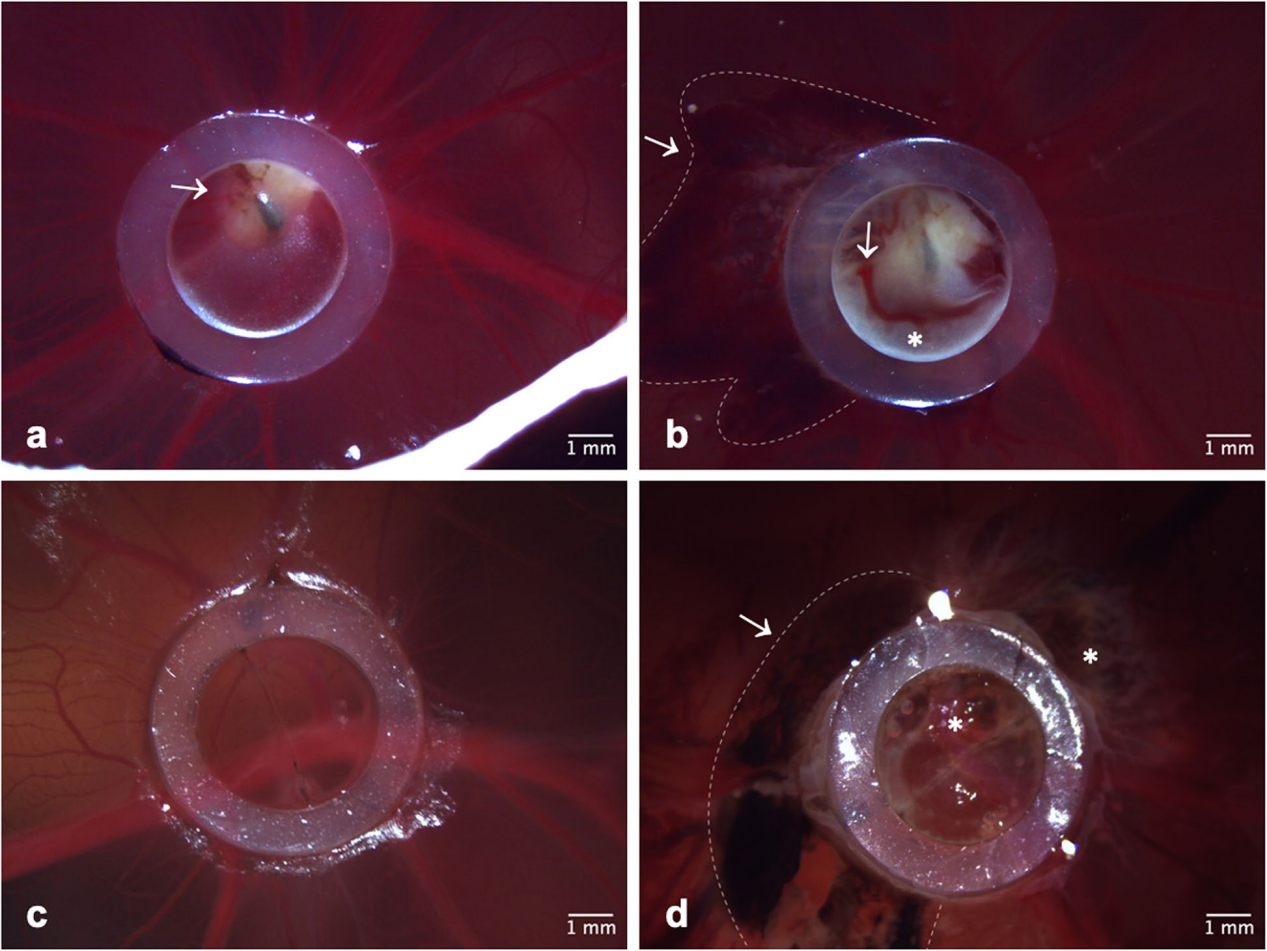
Stereomicroscopic observations. **a** Tumor cell formation (arrow) is visible inside the silicone ring on the CAM on ED 12 before RFA. **b** Signs of heat damage (*) and bleeding (arrow, bordered by dashed line) can be seen 2 h after ablation on ED 12. No tumor cells are visible on a chick embryo of the control cohort before the RFA on ED 12 (**c**), while signs of bleeding (arrow, bordered by dashed line) and heat damage (*) are displayed 2 h after ablation (**d**). *CAM* Chorioallantoic membrane, *ED* Embryonic day, *RFA* Radiofrequency ablation

Common stereomicroscopic observations are given in Fig. 2. Regular findings after ablation were signs of bleeding and signs of heat damage. Furthermore, vaporization was observed on one CAM in the control cohort.

### (Immune-)histological observations

**Fig. 3:**
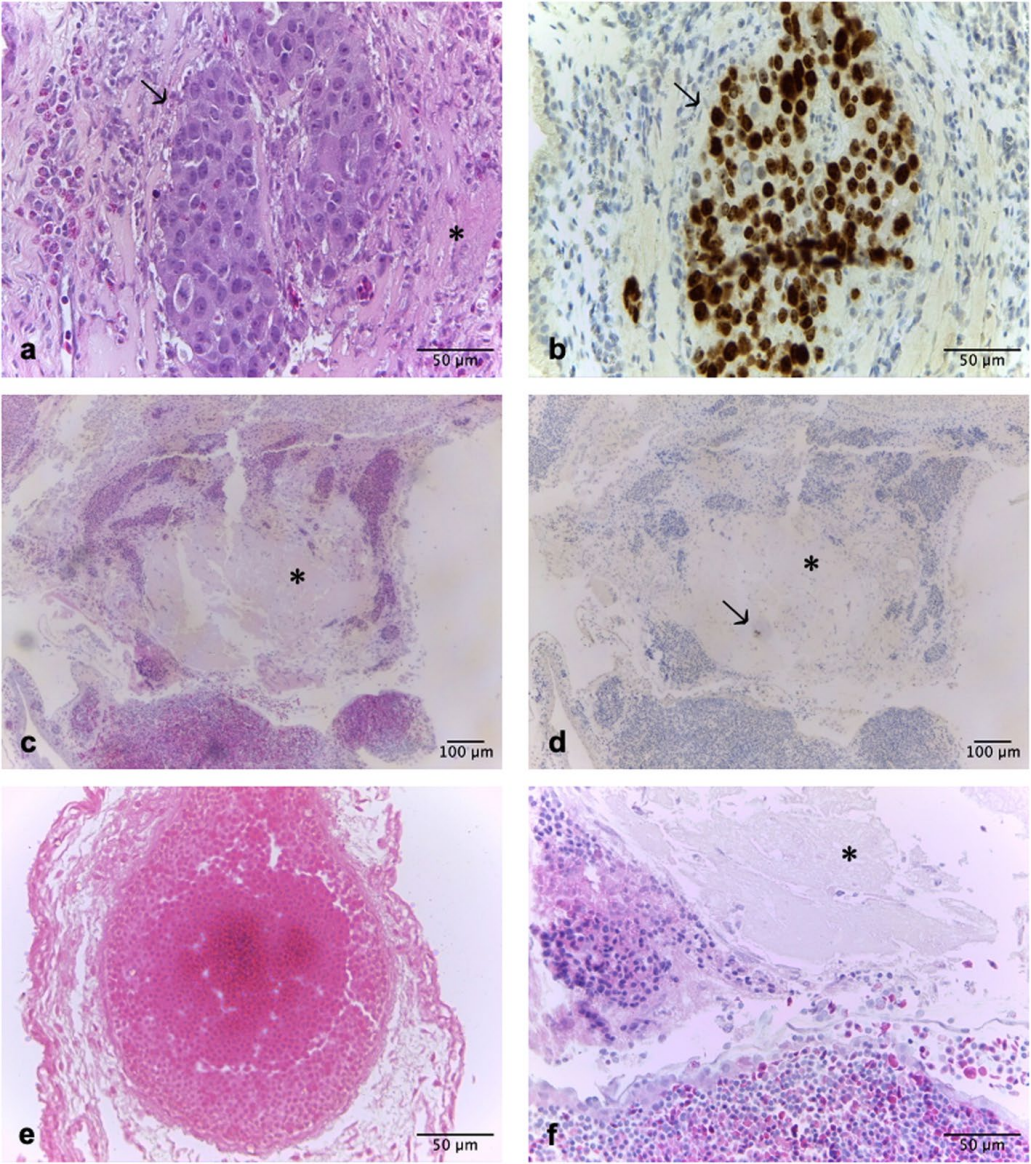
(Immune-)histological observations. **a** and **b** display a cluster of BON-1 cells (arrow) in 40 × magnification H&E staining (**a**) and Ki67 IHC (**b**) in a chick embryo on which no ablation was performed on. Vital BON-1 cells appear larger and more basophilic in H&E staining (**a**) than surrounding tissue and Matrigel (*) and also show a high nuclear to cytoplasmatic ratio. Proliferating tumor cells show brown nuclei in Ki67 IHC, while surrounding chicken cells are negative because the antibody does not bind to chicken Ki67 (**b**). **c** depicts the overview of an ablated chorioallantoic membrane (CAM) in H&E (10 × magnification). In the corresponding Ki67 staining, a single Ki67-positive cell (arrow) can be seen within the Matrigel (**d**, 10 × magnification). Common findings after RFA were thrombosis (**e**) and the presence of leukocytes and erythrocytes indicating inflammatory response with leukocyte infiltration (**f**). Black scale bars in **a**, **b**, **e**, and **f** (40 × magnification) indicate 50 μm, while black scale bars in **c** and **d** (10 × magnification) indicate 100 μm. *CAM* Chorioallantoic membrane, *ED* Embryonic day, *H&E* Hematoxylin–eosin, *IHC* Immunohistochemistry, *RFA* Radiofrequency ablation

Histological examination including Ki67 staining and H&E staining was successfully performed in all but one embryo. The analysis of (immune-)histological outcomes was not performed in one embryo because of insufficient histological sections. Heat-induced tissue damage (Fig. 3c, d), bleeding, thrombosis (Fig. 3e), and leukocyte infiltration and hyperemia (Fig. 3f) were common findings in H&E stained cuts.

## Discussion

The presented study provides new insights into the CAM model as a preclinical research model for tumor RFA. The outcomes in the sham and control cohort show the validity of the CAM model for tumor RFA studies. There was no Ki67 staining in the control cohort showing no proliferating cells, while Ki67 staining could be observed in every slice in the sham cohort, indicating that Ki67 can be used to differentiate between tumor cells and nontumor cells in this research model. Reduction but not eradication of Ki67 staining was regularly achievable in this *in ovo* model. Therefore, the CAM model may be suited as a preclinical model for incomplete ablations or for complete ablations after optimization. Furthermore, a survival of 100% in the not ablated sham cohort indicates that there is a low likelihood of a coincidental embryo death.

With the CAM model as a potential model for incomplete ablations, it can be tested if the combination of RFA and other therapies such as local immunotherapy or chemotherapy improves treatment efficacy and completes an otherwise incomplete ablation. This is especially important as incomplete tumor ablations are known to be associated with increased metastatic potential as well as resistance to immunotherapy and chemotherapy [8, 9, 25].

To increase the survival of chicks and to make a complete or incomplete ablation — depending on what is desired — as reliable as possible, there is a need to optimize this model. To optimize this model, it is important to notice that with increased duration of ablation, the completeness rate increased, while the survival decreased. Therefore, the chick embryo should be exposed to as little heat as possible while ensuring sufficient heat supply to tumor cells on the CAM. A possible solution for this could be the fractionation of heat since it is known that temperature > 60 °C leads to almost instant cell death [26]. This would give the chick time to cool down between RFA fractions. Furthermore, a change in survival and completeness is also expected to be achievable through adaption of power output.

Stereomicroscopic observations showed typical findings of thermal ablation. Heat damage as well as local bleedings are expected outcomes in percutaneous ablation. With the CAM as a highly vascularized area, corresponding findings were to be expected [27]. Vaporization is another well-known finding after RFA. It can restrict energy transmission and is considered to occur in temperatures > 105 °C in RFA [26]. Histological findings in this study like destruction of vessels and thrombosis (Fig. 3e) as well as leukocyte infiltration and hyperemia (Fig. 3f) match literature on histological findings after RFA in humans and also animals using the example of the pig [10, 28]. This suggests that the CAM model is suited to evaluate outcomes of tumor RFA due to sufficient histological comparability. The presence of these typical histological changes and the fact that the avian immune system is functionally comparable to the human immune system further support the analogy of this *in ovo* model to other *in vivo* models and therefore also the use for preclinical studies [29].

The CAM model for tumor RFA allows the observation of complete and incomplete eradication of Ki67 staining with the possibility to use incomplete ablations as a chance for studies on combined therapies. The outcomes of the sham and control cohort demonstrate validity of the model, while immunohistochemical examinations show that the model is suited for histological evaluation. Furthermore, this model compares favorably to other animal models due to the chicken embryos non-sentience, its low cost, simplicity, reproducibility, and short duration of experimentation [13, 30]. Moreover, there is no need for an approval of an ethics committee in many countries which allows a fast start of experimentation after the planning is finished [20].

This study is limited by the small number of ablated chicken embryos. Nevertheless, the feasibility of the CAM model for tumor RFA could be shown in this study. The number of chicken embryos experimented on was intentionally small to reduce the number of animals in concordance with the “Three Rs” recommended for experimental techniques [7].

The CAM model is a feasible and suiting research model for tumor RFA with many advantages over other animal models. It offers the opportunity to conduct *in vivo* research under standardized conditions. However, further studies are needed to optimize this model for tumor ablations in order to explore promising but unrefined strategies like the combination of RFA and immunotherapy.

## Data Availability

The data that support the findings of this study are available from JJ upon reasonable request.

## Abbreviations

CAM: Chorioallantoic membrane
ED: Embryonic day
H&E: Hematoxylin-eosin
IHC: Immunohistochemistry
RFA: Radiofrequency ablation
